# Noninvasive hemoglobin sensing and imaging: optical tools for disease diagnosis

**DOI:** 10.1117/1.JBO.27.8.080901

**Published:** 2022-08-03

**Authors:** Michaela Taylor-Williams, Graham Spicer, Gemma Bale, Sarah E. Bohndiek

**Affiliations:** a University of Cambridge, Department of Physics, Cavendish Laboratory, Cambridge, United Kingdom; b University of Cambridge, Cancer Research UK Cambridge Institute, Cambridge, United Kingdom; c University of Cambridge, Electrical Division, Department of Engineering, Cambridge, United Kingdom

**Keywords:** hemoglobin, sensing, imaging, spectroscopy

## Abstract

**Significance:**

Measurement and imaging of hemoglobin oxygenation are used extensively in the detection and diagnosis of disease; however, the applied instruments vary widely in their depth of imaging, spatiotemporal resolution, sensitivity, accuracy, complexity, physical size, and cost. The wide variation in available instrumentation can make it challenging for end users to select the appropriate tools for their application and to understand the relative limitations of different methods.

**Aim:**

We aim to provide a systematic overview of the field of hemoglobin imaging and sensing.

**Approach:**

We reviewed the sensing and imaging methods used to analyze hemoglobin oxygenation, including pulse oximetry, spectral reflectance imaging, diffuse optical imaging, spectroscopic optical coherence tomography, photoacoustic imaging, and diffuse correlation spectroscopy.

**Results:**

We compared and contrasted the ability of different methods to determine hemoglobin biomarkers such as oxygenation while considering factors that influence their practical application.

**Conclusions:**

We highlight key limitations in the current state-of-the-art and make suggestions for routes to advance the clinical use and interpretation of hemoglobin oxygenation information.

## 1 Introduction

Optical-imaging biomarkers are defined characteristics measured with an optical imaging modality to indicate normal biological or pathological processes. Optical-imaging biomarkers can help researchers better understand disease development and give clinicians the ability to diagnose and treat diseases in patients.^1^^,^^2^

Based on the absorption of light by hemoglobin, optical imaging biomarkers such as hemoglobin concentration, oxygen saturation, and blood flow can be measured with a range of instruments for clinical disease evaluation. Hemoglobin is a protein in blood that transports oxygen to organs. Oxygen plays a vital role in cellular aerobic respiration, where it reacts with glucose to form adenosine triphosphate, water ($H_{2}O$), and carbon dioxide (${\mathrm{CO}}_{2}$), essential for maintaining healthy tissue and blood vessels. Hemoglobin oxygenation is often used as a vital sign; low oxygenation at the level of the organism can indicate a systemic disease, such as chronic obstructive pulmonary disease and apnea.^3^^,^^4^ Poor oxygenation in a particular organ or tissue can be symptomatic of an insult due to injury or illness, such as diabetes, skin trauma, rheumatic disease, or cancer.^1^^,^^5^^–^^8^

Noninvasive, low-cost, safe, and portable methods based on optics for extracting hemoglobin-derived biomarkers have become vital tools in patient management that can be applied in real time at the bedside. Despite widespread use of methods such as pulse oximetry, the uptake of newer technologies that go beyond point measurements remains relatively limited; however, there are many promising tools in development, ranging from 3D volumetric imaging of vascular architecture to spatially-resolved functional images of tissue oxygenation. Being less expensive and more portable in general than conventional radiological imaging methods, these have the potential to impact patient care in a wide range of debilitating illnesses, ranging from rheumatoid and vascular diseases to neurodegenerative diseases and cancer.

Here, we review these noninvasive methods for quantifying hemoglobin-derived biomarkers, including pulse oximetry, as commonly used in clinical practice worldwide, together with promising tools emerging in the research setting for imaging. The relative strengths and weaknesses of different methods are considered according to the application, grouped by mode of operation, including single-point detection, superficial imaging (up to 1- to 2-mm depth); and deep tissue imaging. We compare techniques based on the technology used, analysis methods, and current research or clinical applications; we then highlight limitations that would benefit from future research.

## 2 Impact of Tissue Properties on Optical Measurement of Hemoglobin Biomarkers

### 2.1 Biology of Human Blood

Human blood consists of plasma (about 55 vol.%) and cells (∼45 vol.%) in which 99% of the cells are red blood cells (RBCs) and the remaining 1% are leukocytes and thrombocytes.^9^^,^^10^ Plasma is a complex composition of dissolved ions (electrolytes), lipids, sugars, and proteins.^10^ RBCs, also known as erythrocytes, have a flat biconcave shape and a mean volume of 90  μm,^3^ ^9^^,^^11^ and they contain about 30 pg of hemoglobin, a globular metalloprotein responsible for oxygen transport throughout the body.^9^^,^^11^ Hemoglobin concentrations range from 134 to 173  g/L in whole blood and 299 to 357  g/L in RBCs, varying according to age, gender, and health status. For example, anemia, cancer, or hereditary hemochromatosis decrease hemoglobin levels in the blood.^9^^,^^12^

Each hemoglobin molecule contains four heme groups that can be bound to oxygen; the unbound state is referred to as deoxygenated hemoglobin, Hb, and the saturated bound state is considered oxygenated and denoted by ${\mathrm{HbO}}_{2}$.^10^ Sometimes the oxygen saturation of arterial blood, ${\mathrm{SaO}}_{2}$, is differentiated from that of peripheral blood, ${\mathrm{StO}}_{2}$, because arterial blood oxygenation should be the same throughout the body. In contrast, peripheral blood has varying oxygenation levels as oxygen is absorbed from the blood by peripheral systems.^11^^,^^13^^,^^14^ Hemoglobin is also able to bind to other molecules forming carboxyhemoglobin, which arises during carbon monoxide inhalation;^15^^,^^16^ sulfhemoglobin, which arises due to the irreversible binding of sulphur in the presence of sulfonamides;^17^ and carbaminohemoglobin, which results from the binding of hemoglobin in venous blood to carbon dioxide.^18^ A further variant state of hemoglobin is methemoglobin,^19^^–^^21^ in which hemoglobin binds iron in the ${\mathrm{Fe}}^{3+}$ state (unlike normal hemoglobin that binds ${\mathrm{Fe}}^{2+}$), which prevents the binding of oxygen. Methemoglobin occurs naturally in blood at ∼1% to 2% concentration,^15^ but it can be elevated due to side effects of medication or environmental factors.^22^ Finally, genetic variants of hemoglobin, such as hemoglobin S, which causes sickle cell anemia, can influence hemoglobin structure and binding properties.^23^ Myoglobin, a chromophore commonly found in muscles, binds oxygen with a higher affinity than hemoglobin.^24^ Myoglobin has a similar spectral response to hemoglobin and may be found in the bloodstream following muscle injury.^24^^,^^25^

### 2.2 Optical Properties of Biological Tissue

Tissue is a complex turbid medium composed of different cell types and protein-rich extracellular matrix, which strongly impact the propagation of light.^11^^,^^26^^,^^27^ Absorption is the transformation of light energy to some other form of energy, such as heat, sound or fluorescence, as light traverses tissue and is quantified by the absorption coefficient, $\mu_{a}$ (${\mathrm{cm}}^{-1}$). Absorption is the primary optical interaction that is exploited to measure hemoglobin biomarkers. In turbid media, scattering is a major contributor to light attenuation and can confound attempts to measure hemoglobin absorption because blood is a highly scattering liquid with strong anisotropy. Scattering refers to a change in the direction of light propagation and is quantified by the scattering coefficient, $\mu_{s}$ (${\mathrm{cm}}^{-1}$), together with directional factors such as the scattering phase function and anisotropy factor, which further relate to the tissue refractive index.

#### 2.2.1 Optical properties of RBCs, hemoglobin, and its derivatives

**Fig. 1:**
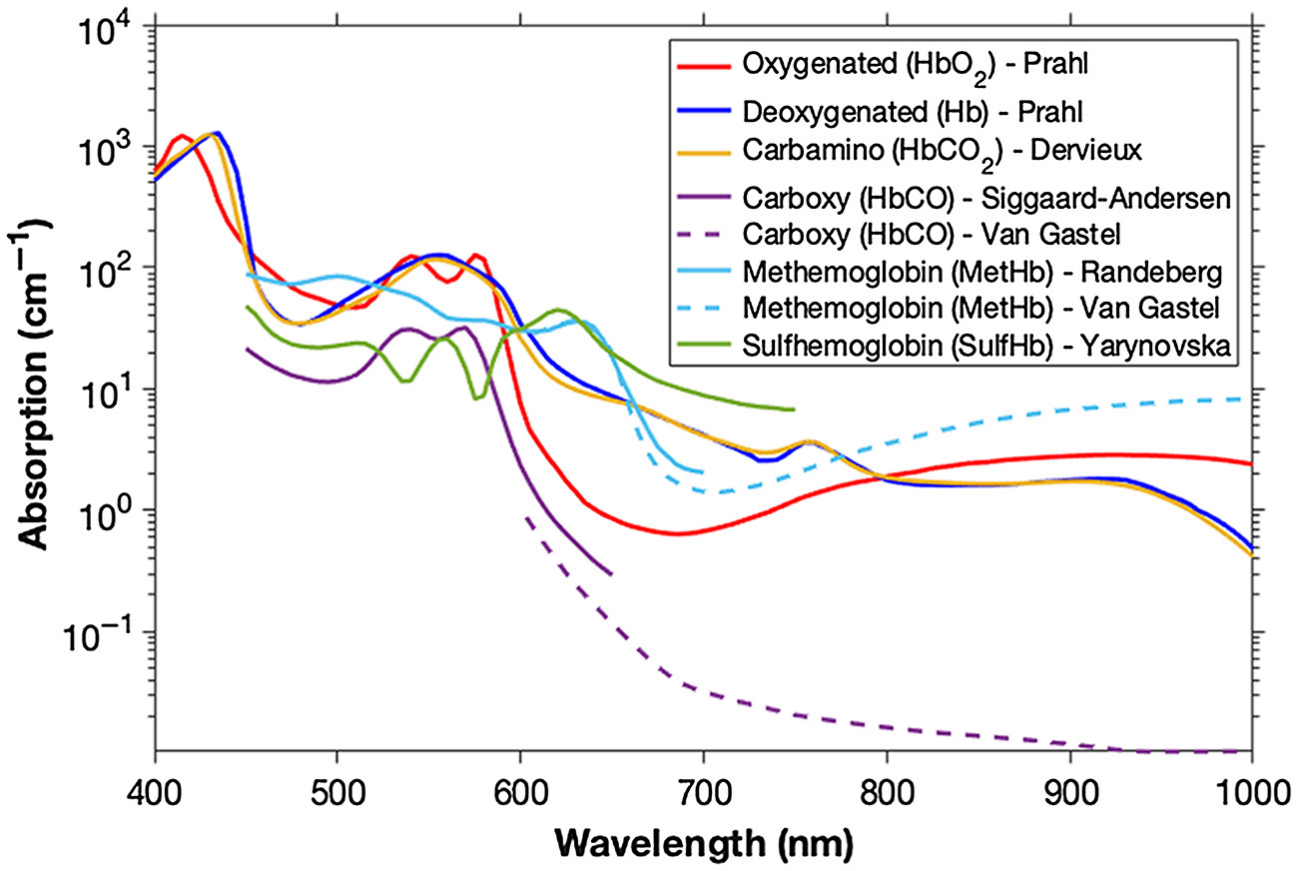
The optical absorption of hemoglobin and associated variants. Representative spectra are shown for oxygenated,^28^ deoxygenated,^28^ carbamino,^18^ carboxy (visible^29^ and NIR^19^^,^^20^), methemoglobin (visible^30^ and NIR^19^^,^^20^), and sulfhemoglobin.^31^

The absorption coefficient of hemoglobin is a function of wavelength and the binding state (Fig. 1).^32^ Hemoglobin is usually oxygen-bound, whereas other variants mentioned above can modify the absorption spectrum and should be considered if relevant to the pathology being assessed because they can confound the measurement.^10^^,^^13^^,^^23^^,^^33^^,^^34^ It should be noted that the spectra of hemoglobin and its variety of physiologically relevant bound states are often measured after extracting the hemoglobin protein from RBCs, so they do not consider variation that arises due to scattering from different RBC geometries and orientations.

In the absence of shear stress, human RBCs are biconcave discs with a diameter of 7 to 8  μm, maximal thickness of 2 to 3  μm, and minimal thickness of 0.8 to 1.5  μm.^35^^,^^36^ Concentrations of Hb within an RBC are high, on the order of 300 to 360  mg/mL, and the total refractive index of the cell is well approximated by that of pure hemoglobin through the application of the Kramers–Kronig relations on the total absorption spectrum.^37^^,^^38^ When light is incident upon a single RBC, scatter from the near and far membrane interfaces leads to a characteristic oscillatory scattering spectrum and phase function.^36^^,^^39^ The oscillatory spectral shape makes oximetry of a single RBC impossible without *a priori* knowledge of the precise shape and orientation of the RBC; however, averaging over many cells with random orientation can smooth this oscillation, which permits measurement of oxygen saturation of RBCs in capillaries.^39^^,^^40^ In addition, hematocrit, the volume fraction of RBCs in whole blood, the presence of blood plasma, and other factors affect the absorption spectra.^18^^,^^41^^–^^44^

The most common hemoglobin-derived optical imaging biomarkers are total hemoglobin (referred to as THb hereafter) and oxygen saturation (referred to as ${\mathrm{sO}}_{2}$ hereafter). THb is often evaluated using a single wavelength absorption measurement taken at an isosbestic point of ${\mathrm{HbO}}_{2}$ and Hb (i.e., when their absorption coefficients are equal). ${\mathrm{sO}}_{2}$ requires an absorption measurement to be made at multiple wavelengths (at least 2), usually spanning regions where either ${\mathrm{HbO}}_{2}$ and Hb dominate the absorption properties. Data are then often analyzed by applying multivariate statistical approaches for spectral unmixing^45^ to extract the ${\mathrm{sO}}_{2}$ value. The absorption coefficients of ${\mathrm{HbO}}_{2}$ and Hb are related through ${\mathrm{sO}}_{2}$ to the overall optical absorption $\mu_{a}$ as^11^^,^^45^

$${\mathrm{sO}}_{2}=\frac{\left[{\mathrm{HbO}}_{2}\right]}{\left[{\mathrm{HbO}}_{2}]+[\mathrm{Hb}\right]},$$ (1)

$$\mu_{a}(\lambda )=c_{\mathrm{Hb}\;}\{\frac{{\mathrm{sO}}_{2}}{100}\;\mu_{{\mathrm{aHbO}}_{2}}(\lambda )+(1-\frac{{\mathrm{sO}}_{2}}{100})\mu_{\mathrm{aHb}}(\lambda )\},$$ (2)

where $c_{\mathrm{Hb}}$ is the concentration of hemoglobin in the tissue.

In addition to hemoglobin, many other molecules interact with light depending on their concentration and distribution throughout the tissue,^46^ which can disrupt light propagation and also introduce spectral coloring at depth in tissue, confounding attempts to measure THb and ${\mathrm{sO}}_{2}$. Furthermore, the scattering and refractive index properties of blood are affected by hemoglobin concentration, erythrocyte volume, shape, and aggregation, each of which can be modified in disease. Unlike the absorption coefficient, the anisotropy and the scattering coefficients of blood are not dependent on the changes in oxygenation.^9^^,^^47^

Hemoglobin-derived biomarkers often rely on variations in the absorption or scattering by hemoglobin molecules or RBCs, and the hardware used to make these measurements varies, including the use of LEDs or lasers for illumination with optical sensors (both arrays and point sensors) or ultrasound sensors for detection. The methods described in this review are summarized in Table 1.

**Table 1:** Overview of noninvasive hemoglobin monitoring and imaging.

Technology	Clinical application(s)	Spatial resolution	Benefits	Limitations	Clinical status and future potential	References
**Pulse oximetry**	Used to identify hypoxemia in a range of healthcare settings.	Single point measurement with no spatial resolution.	•Real-time monitoring of arterial saturation. •Extremely simple and quick to use in a clinical setting. •It can be used at the patient’s bedside.	•Single point location measurement. •It can be inaccurate due to calibration assumptions •Errors are associated with variations in hemoglobin and poor perfusion on the tissue measured.	Commonly used in primary through to tertiary care. Potential for increased deployment at-home through wearables and low-cost devices.	13,48–59
**Nailfold capillaroscopy**	Diagnosing and monitoring systemic scleroderma and other arthritic conditions.	Spatial resolution varies from 0.1 to 10 μm laterally dependent upon the imaging NA, with corresponding imaging focal depth (Rayleigh range).	•Real-time imaging of capillaries and blood flow is relatively easy. •Typically, the design is portable, so it can be used at the bedside if needed.	•Lack of standardization around the quantification of capillary parameters. •Measures only structural, not functional, information about the capillaries. •Restricted to only imaging the nailfold capillaries.	Used in tertiary care. Emerging methods include quantification of blood flow, blood cell counts, and oxygenation imaging.	5,60–70
**Spectral reflectance imaging (MSI and HSI)**	Narrowband endoscopic imaging.	Spatial resolution varies from 5 to 400 μm depending on the application because most systems use lenses for magnification. Reflectance signal restricted to superficial to 200 μm of tissue due to scattering.	•A versatile method that can image multiple hemoglobin biomarkers as well as other proteins of interest. •Relatively high spatial resolution is possible.	•Processing of images can be complicated and is not always possible in real time •Snapshot methods are fast but sacrifice spectral and spatial resolution •Scanning methods are slow but have a higher spectral resolution.	Commonly used in tertiary care for endoscopic surveillance. Otherwise, used in research and small-scale clinical trials with promising applications in cancer detection, skin lesions, e.g., burns, and surgical tissue health.	2,6,8,30,71–85
**PAI**	Breast lesion evaluation.	PAM can have a spatial resolution of ∼30 μm with an imaging depth of around 2 to 6 mm. PAT can have a resolution of ∼200 μm with a penetration depth 2 to 3 cm.	•Can assess the relative hemoglobin saturation together with other biomarkers at depth while maintaining a reasonable spatial resolution. •It is a versatile technique scaling spatial resolution with depth of imaging required.	•Trade-off between resolution and depth. •Requires acoustic contact between the tissue and detectors. •Systems can be costly, though LEDs can be used at the expense of imaging quality. •The use of high-power pulsed light can present additional safety considerations to ensure that stray light does not damage patients or clinicians’ eyes	Large scale clinical trials (n>2000) for the diagnosis of breast cancer. Small scale clinical trials or proof of concept for other applications, such as severity assessment in Crohn’s disease and dermatological conditions.	32,86–109
**Spectroscopic OCT**	Structural OCT is a clinical standard of care for retinal imaging. Spectroscopic OCT not yet clinically approved.	Lateral resolution determined by illumination optics, typically tens of μm but as low as 1 to 2 μm is achievable. Axial resolution determined by source/detector bandwidth, on the order of tens of μm down to 1 μm. Penetration depth is limited to ∼1 mm in medium-scattering tissue.	Real-time tomographic imaging with high resolution ideal for resolving tissue layers, structural characteristics in 3D.	•Optical scattering in tissue limits imaging depth to 1 to 2 mm for most tissues. •Laser scanning of sample introduces potential for motion artifacts in image, ophthalmic visible OCT implementation limited by safety threshold and patient aversion.	Structural OCT widely used in primary and tertiary centers for ophthalmology, dermatology, and dentistry, also in tertiary centers for cardiology. Spectroscopic OCT for oximetry in preclinical development.	39, 110–122
**DOI or NIRS**	Assessment of brain activity (fNIRS or DOT). Monitoring of tissue oxygenation (cerebral oximetry/NIRS).	1 to 30 mm spatial resolution; high resolutions are only possible at shallow imaging depths (<2 cm). Low-resolution imaging is possible up to 10 cm into the tissue.	Capable of determining blood oxygenation in tissue and other chromophores such as melanin, lipids, cytochrome-c-oxidase, and water.	•Relatively low resolution; obtaining high resolution requires prior knowledge of tissue composition and a high density of optodes. •DOI techniques may be combined with other imaging modalities such as MRI or ultrasound to assess tissue composition, resulting in increased system cost. •Computationally-intensive and time-consuming, resulting in limited real-time imaging (spectroscopy does not have this problem).	Clinical approval and small-scale clinical use of tissue oximetry/NIRS for assessment of brain oxygenation. fNIRS/DOT used commonly as a research tool to monitor brain activity. Small scale clinical trials or proof of concept for other applications, such as breast cancer diagnosis, and joint inflammation.	123–134

## 3 Point Sensing of Hemoglobin ${\mathrm{sO}}_{2}$ through Pulse Oximetry

Pulse oximetry makes a localized measurement of arterial hemoglobin ${\mathrm{sO}}_{2}$. To make this measurement, the absorption of tissue is evaluated at two or more different wavelengths, selected according to where the absorption coefficients of Hb and ${\mathrm{HbO}}_{2}$ differ sufficiently for their ratio to be evaluated as a biomarker that can be correlated directly to ${\mathrm{sO}}_{2}$ [Eq. (1)].

### 3.1 Clinical Applications and Research Studies

Pulse oximetry has been extensively reviewed elsewhere.^13^^,^^48^^,^^49^^,^^135^^,^^136^ Pulse oximetry is deployed in many medical applications, from at-home first aid to clinical intensive care units and surgical theaters^13^, and it has found particular utility in assessing hypoxemia in COVID-19 patients^137^ as nonspecialists with minimal training can efficiently operate pulse oximeters.^49^^,^^138^ Pulse oximetry research in the clinic focuses on its use in treating and diagnosing diseases, such as optimizing oxygenation of ventilated patients, screening neonates for congenital heart diseases, and monitoring patients with sleep apneas.^50^^,^^51^^,^^139^^,^^140^ Despite widespread use, it is also well established that pulse oximetry can suffer racial bias, which results in less accurate oxygenation readings for patients with more skin melanin content, a trait associated with darker skin. The impact of such bias is severe as it has been shown to result in less adequate medical treatment of such patients, meaning that it is important for clinicians to be aware of this limitation and it is also an important area for future research and development.^141^^,^^142^ Although this bias has been well known for some time, it has been increasingly studied as a result of COVID-19 and the increased clinical use of pulse oximeters to treat respiratory conditions.

### 3.2 Technology

Light absorption in pulse oximetry is typically measured using alternating illumination by LEDs at two different wavelengths.^135^^,^^143^^,^^144^ Because the wavelength of the illuminating light is altered with time, oxygenation measurements are susceptible to motion artifacts, which change the area of tissue being illuminated and coupling to the tissue, resulting in inaccuracies. Commonly used wavelength pairs are 660 and 940 nm or 665 and 894 nm,^13^^,^^52^^–^^55^ which are applied in two different modes.

**Fig. 2:**
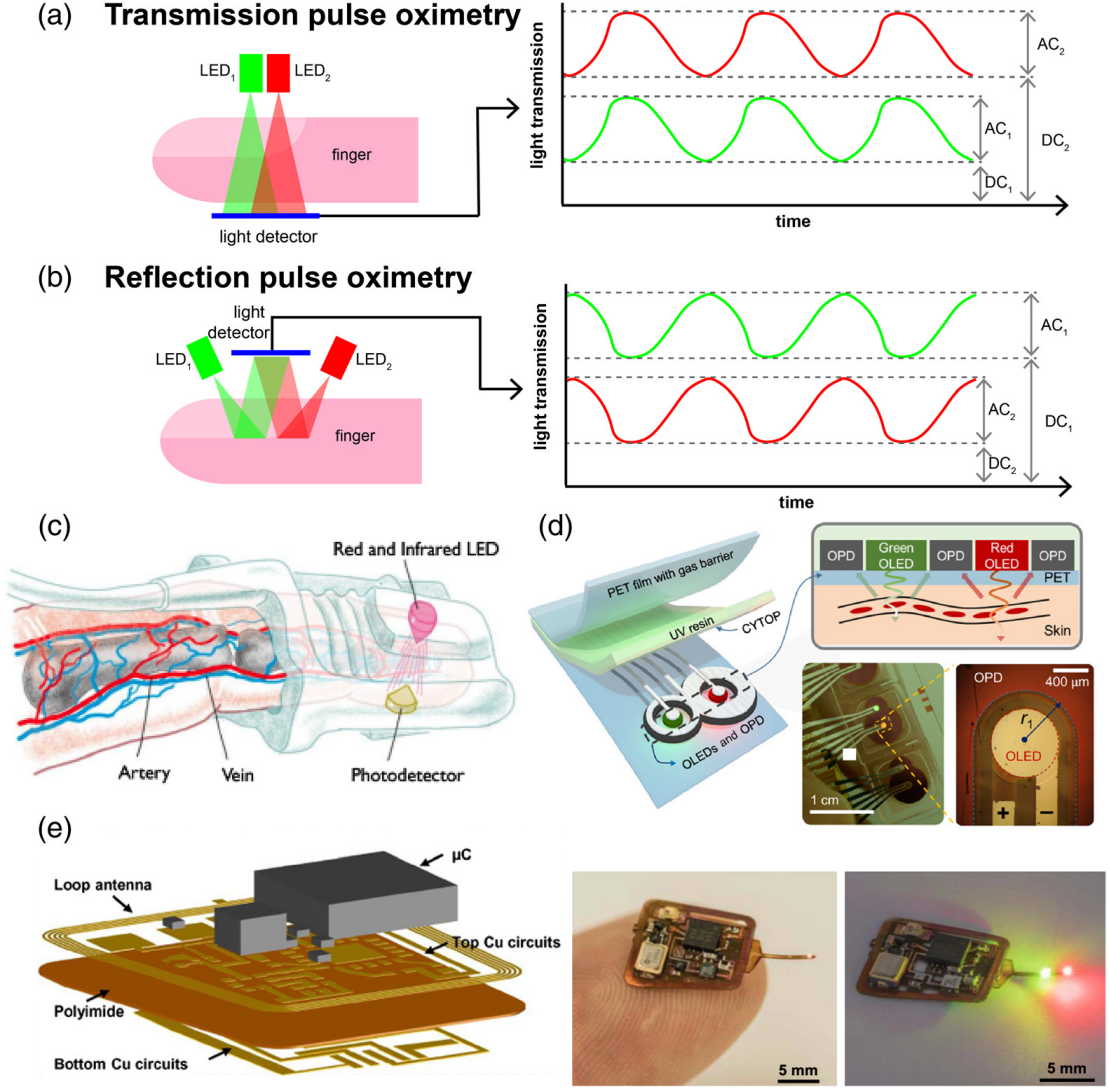
Pulse oximetry. Schematic illustration of pulse oximetry in the two different operation modes: (a) transmission and (b) reflection. The detected light is cyclic due to the pulsatile nature of blood in the peripheral vascular system. Both transmission and reflection modes have alternating components (AC) and direct components (DC). In tissue, the transmission and reflection of light vary based on the changes in absorption due to blood volume and oxygenation. That is R+A+T≈1, when R, A, and T are the normalized reflection, absorption, and transmission intensities, respectively. For this reason, in reflection pulse oximetry, the peak intensity of light will be off by half a cycle from that of the transmission cycle. Examples of pulse oximeter devices include (c) transmission-based devices widely used in a clinical setting. Reproduced with permission from Ref. 145. (d) Low-power devices in development that adhere to the skin and use flexible OLED illumination. Reproduced with permission from Ref. 51. (e) Battery-free pulse oximeters in development that use near field communication for power. Reproduced with permission from Ref. 146.

• Transmission: Tissue such as the finger, toe, or earlobe is illuminated, and the light transmitted through the tissue is detected by a sensor to determine the amount of light attenuated by the tissue [Fig. 2(a)].
• Reflection: Tissue such as the finger, foot, or forehead is illuminated, and the amount of light reflected by the tissue and underlying bone is detected by a sensor and used to determine the amount of light that the tissue has absorbed. Reflection pulse oximetry tends to have a higher signal when there is low perfusion.^13^^,^^147^ In reflection pulse oximetry, it can be challenging to isolate the light that has gone deeper into tissue from light that has been scattered or reflected at the surface of the tissue [Fig. 2(b)].

Evolving from the traditional fingertip pulse oximeters [Fig. 2(c)], the current development of the technology mainly targets wearable devices and focuses on low power usage [Fig. 2(d)], optimization of signal detection, reduction of motion artifacts, flexible illumination and detection [Fig. 2(e)], low-cost devices, miniaturization, and calibration techniques.^51^^,^^56^^–^^59^

### 3.3 Analysis

The theory of oximetry analysis has been extensively reviewed by Mackenzie and Harvey,^148^ so it will be only briefly introduced; readers are referred to the prior review for a more detailed description. The total extinction coefficient for blood is denoted as ε and related to ${\mathrm{SaO}}_{2}$ as^13^

$$\varepsilon =\varepsilon_{O}{\mathrm{SaO}}_{2}+\varepsilon_{D}(1-{\mathrm{SaO}}_{2}).$$ (3)

Further analysis of the extinction coefficient is needed to isolate the signal from arterial blood because venous blood also absorbs the light, along with other chromophores that appear in the light path, such as melanin (in the skin). It is possible to exploit the cyclic nature of the extinction coefficient due to the pumping of blood by finding the ratio of the variable component (AC) and constant component (DC) at two different wavelengths [Figs. 2(a) and 2(b)], where the difference in light absorption is rather large^13^^,^^48^^,^^135^

$$R=\frac{{\left(\mathrm{AC}/\mathrm{DC}\right)}_{1}}{{\left(\mathrm{AC}/\mathrm{DC}\right)}_{2}}.$$ (4)

The ratio R, also known as the modulation ratio, is then related to ${\mathrm{SaO}}_{2}$ through a calibration procedure using best-fit analysis according to the equation

$${\mathrm{SaO}}_{2}=a+bR,$$ (5)

where the variables a and b are calculated for each device during testing, based on a linear regression between the modulation ratio and the ${\mathrm{SaO}}_{2}$ value.^135^^,^^149^ Calibration was originally performed with human volunteers, changing ${\mathrm{SaO}}_{2}$ values by limiting the oxygen in the air that they breathed from 70% to 100% ${\mathrm{SaO}}_{2}$, which determined the R values.^150^ These are valid across devices with the same design, which means that individual devices did not have to be calibrated. Calibration techniques have evolved, so volunteers are no longer required. For example, several devices simulate the circulatory system and finger using pumps to mimic the pulsatile flow of arterial blood and venous blood.^12^^,^^151^^–^^155^ The system is then sealed off, and the oxygenation of the blood can be controlled by varying the oxygen content of the system. Alternatives include electrical simulators that emit light from an LED corresponding to the light detected by the sensor to mimic the light transmitted in a typical finger. This technique requires prior knowledge of the pulse oximeter being calibrated. Once in operation, pulse oximeters are rarely recalibrated.^150^

### 3.4 Limitations

Pulse oximetry measurements typically have an error of 3% to 4% depending on the device and calibration used, which is actually sufficient to impact patient care in some cases.^150^^,^^156^ In addition, standard pulse oximeters cannot detect the presence of methemoglobin, carboxyhemoglobin, or hemoglobin mutations, although their presence and concentrations outside of the expected range will affect the oxygenation readings.^15^^,^^23^ Some targeted pulse oximeters are able to detect methemoglobin and carboxyhemoglobin, but they are usually used in specific scenarios in which high levels of these derivatives are expected due to exposure.^157^ Hemoglobin F, present in fetuses and infants under 6 months, has a greater oxygen affinity, which allows the fetus to absorb oxygen from the mother’s bloodstream;^10^ in infants, its presence can increase the error in pulse oximetry by a further 3% in addition to the typical errors.^13^ Additionally, when there is poor perfusion to tissues, pulse oximetry can be limited, and if there is not a significant pulse detected, the technique will have increased inaccuracies.^55^ Finally, pulse oximetry has been found to suffer racial bias in two large cohorts, in which black patients had nearly three times the frequency of occult hypoxemia not detected by pulse oximetry as white patients.^141^^,^^142^ Skin pigmentation leads to an overestimation of arterial oxygen saturation in dark-skinned individuals, which could seriously impact medical decision making and long-term outcomes.^158^ These limitations merit increased attention in research and development given the potential for long-term and widespread use of pulse oximetry in COVID-19 patient management and the interest in deployment of the technology in the wearable setting.

## 4 Reflectance Imaging of Hemoglobin

**Fig. 3:**
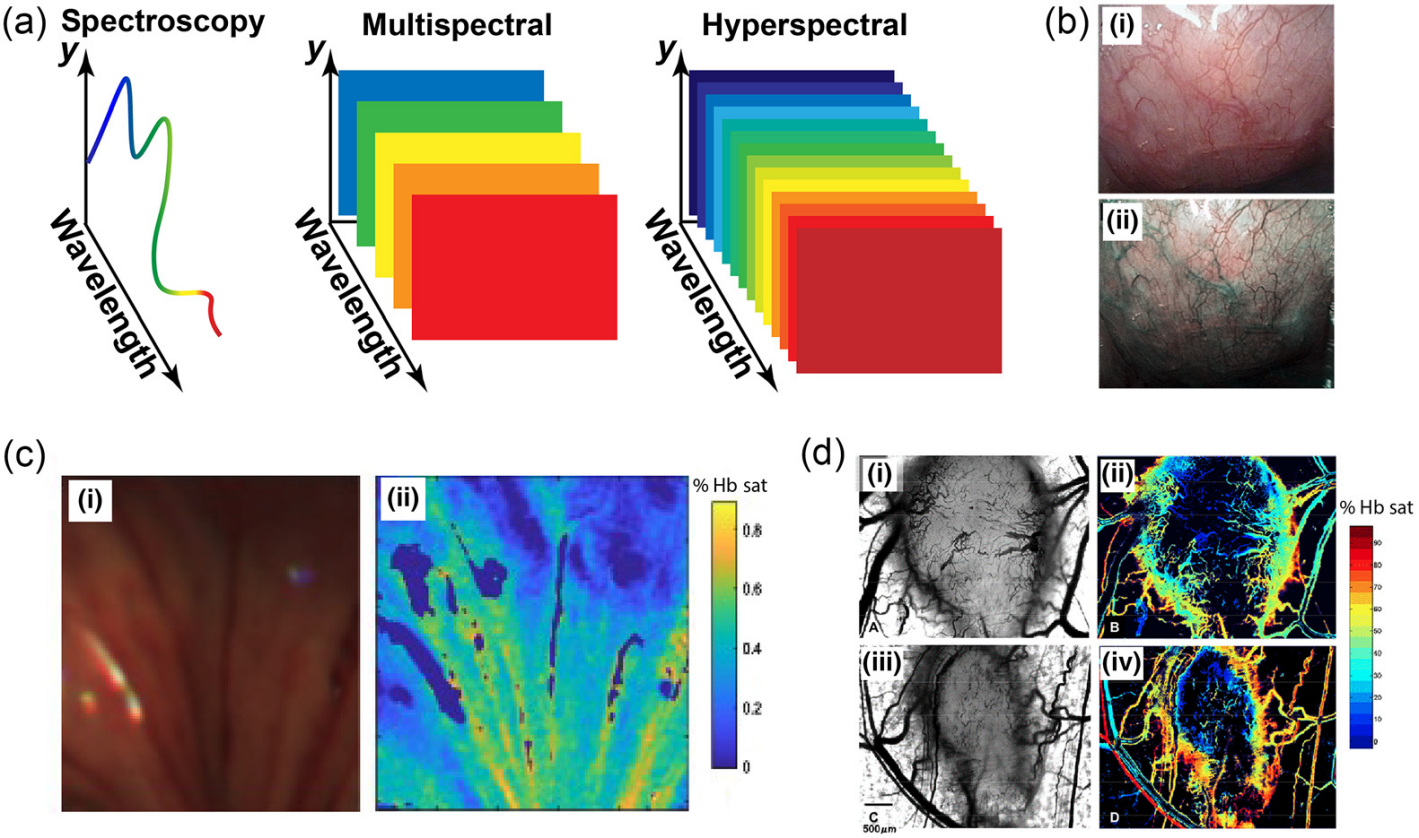
Spectral reflectance imaging. (a) Overview of spectral reflectance imaging methods.^159^ Point-scanning spectroscopy can be used to build spectral information using a standard spectrometer. Alternatively, a spectral camera can be used to collect either a limited number of wavelengths (multispectral, typically <10 spectral bands) or a more continuous spectrum (hyperspectral). (b) Endoscopy images of the esophagus with (i) RGB imaging and (ii) narrowband imaging, which improves the contrast of the blood vessels. Reproduced with permission from Ref. 160. (c) Endoscopy of a porcine esophagus to determine tissue viability with 24 spectral bands from 460 to 690 nm (spectral resolution of 10 nm) using a slit hyperspectral imaging and fiber bundle probe and the resulting (i) reconstructed RGB image and (ii) unmixed oxygenation. Reproduced with permission from Ref. 161. (d) Hypoxia of tumors can be imaged using a liquid crystal tunable filter in conjunction with a CCD; this is demonstrated in mouse tumors; (i) and (iii) light microscopy of tumor vasculature in a dorsal skin window chamber, and the additional information of hemoglobin saturation is shown in (ii) and (iv) illustrating low oxygen saturation of the tumors. Reproduced with permission from Ref. 162.

Optical imaging of hemoglobin biomarkers requires the operator to build a spatially resolved map of hemoglobin absorption at multiple wavelengths, again exploiting the differential absorption coefficients of Hb and ${\mathrm{HbO}}_{2}$.^26^^,^^71^^,^^159^ Several methods can be used to achieve this, including point-scanning spectroscopy, multispectral imaging, and hyperspectral imaging [Fig. 3(a)]. The result is a 3D dataset ((x,y,λ))^26^^,^^72^^,^^159^^,^^163^ that can be subjected to multivariate analysis methods to extract from the measured spectra the concentrations of their contributing chromophores (e.g., Hb and ${\mathrm{HbO}}_{2}$), referred to as “endmembers” for unmixing.^26^^,^^72^^,^^164^^–^^167^ From these multivariate analyses, biomarkers that relate to THb and ${\mathrm{sO}}_{2}$ can then be extracted.

### 4.1 Clinical Applications and Research Studies

A widespread application of reflectance hemoglobin imaging is in gastrointestinal endoscopy. Virtual chromoendoscopy methods adapt the light source of the endoscopy to focus on two wavelength bands (415 and 540 nm), where hemoglobin absorbs strongly (Fig. 1), thus providing a high contrast morphological image of the tissue vasculature to the clinician.^2^^,^^168^ Narrowband imaging is the most widely established of these methods and meets the ASGE thresholds for targeting biopsies when imaging patients with Barrett’s esophagus for early signs of cancer.^169^ More recently, clinical research studies have demonstrated that, by expanding the number of wavelengths captured in endoscopy,^72^^,^^73^^,^^170^ it is possible to derive hemoglobin biomarkers of THb and ${\mathrm{sO}}_{2}$ from spectral information to classify disease status;^171^ however, further clinical study is needed to demonstrate efficacy.

Capillaroscopy is another reflectance-based imaging technique; it is used to image the blood vessels in the finger nailfold to diagnose disease, particularly to identify rheumatic diseases such as systemic scleroderma. Capillaries are microblood vessels from which oxygen and other nutrients are exchanged with the surrounding cells. In the finger nailfold, the capillaries are oriented in loops parallel to the skin, allowing for full visualization at high resolution in reflectance imaging mode. Capillary walls, formed from a single layer of endothelial cells lining the vessels, can rarely be detected during capillaroscopy, whereas the RBC column is visible, and morphological features associated with the capillary can be measured using monochrome, narrowband, and RGB imaging.^60^ Capillary blood flow in the finger ranges in velocity from 0.67 to 4  mm/s depending on physiological factors and the cyclic nature of perfusion.^172^^–^^174^ Morphological dysfunction of the capillaries is easily identified using the current techniques, but current methods do not make oximetry measurements related to this dysfunction.

Reflectance-based oximetry imaging has been widely explored in retinal imaging because the retina is one of the most metabolically active tissues in the human body.^175^ Commercial retinal oximeters are applied to fundus cameras and use dual-wavelength illumination, akin to pulse oximetry, to acquire images simultaneously at one isosbestic wavelength and one sensitive to ${\mathrm{HbO}}_{2}$. Abnormal retinal oxygenation has been shown to detect diabetic retinopathy, age-related macular degeneration, and glaucoma.^74^^,^^75^^,^^176^ In addition, the retina has similar vascular properties to the brain, making it a perfect window for understanding and diagnosing neurological disease in addition to ocular disease.^75^ Nonetheless, retinal oximetry has yet to find routine clinical application, limited primarily by the impact of fundus pigmentation and vessel size on quantification.^75^ Although some hyperspectral imaging technologies have been explored in an attempt to overcome these limitations, to the best of our knowledge, they have not found clinical application.

In smaller scale studies, clinical trials of reflectance-based hemoglobin imaging have been applied in areas from the skin to the brain. For example, the hemodynamic response of the human cortex has been visualized during open-cranium surgery using hyperspectral imaging combined with multivariate analysis.^76^ Evaluation of hemoglobin oxygenation and melanin content in the face has been of interest for dermatological treatments such as the detection of skin cancer, assessment of scar healing, and evaluation of skin thickness.^177^^–^^180^ Spectral imaging of hemoglobin in burns,^8^ wounds,^6^ and bruises^30^^,^^77^ has been explored to assess the progress of healing in a quantitative manner. Moreover, spectral imaging of tissue ${\mathrm{sO}}_{2}$ has found application in intraoperative imaging.^161^^,^^181^ Postoperative imaging can also provide clinicians with information on how tissue is healing such as following breast reconstruction in which water, hemoglobin concentration, and oxygen saturation are key indicators for tissue perfusion, an important factor in recovery.^182^

### 4.2 Technology

The simplest imaging oximetry methods include two or three wavelengths akin to pulse oximetry, which can be applied using sequential illumination by the target wavelength bands and imaging with a single camera or simultaneous illumination of all wavelengths (e.g., with broadband illumination) and capture using a spectrally resolved method, such as image splitting through band pass filters applied in front of two cameras. Expanding the spectral range of wavelengths to capture more spectral features of Hb or ${\mathrm{HbO}}_{2}$ (in the visible but also near-infrared range^183^) requires more complex hardware.

There are three main types of multi or hyperspectral systems used in medical imaging that can be applied for unmixing oxygenation of tissue.^78^^,^^79^^,^^159^ In spatial scanning, a spectrograph records the spectral dimension (λ) while being scanned across a sample. A one-dimensional spectrograph may be point-scanned or a two-dimensional (2D) spectrograph may be line scanned. The approach sacrifices temporal resolution and hence requires minimal movement of the sample to avoid motion artifacts. In spectral scanning, a 2D camera records the spatial dimensions (x,y), while the spectral dimension is scanned, e.g., by changing the illumination wavelength or by filtering the imaging light path (e.g., with a filter wheel or tunable filter). This also has a scanning time associated with it, so it requires minimal movement to prevent spectral artifacts.

Finally, in snapshot methods, a system outputs spectral and spatial dimensions (x,y,λ) simultaneously without scanning.^78^^,^^79^ Snapshot systems may use beam splitting with dichroic filters, volume holographic optical element splitters, image replicating spectrometers, or multispectral filter arrays in the imaging path. Snapshot methods avoid motion artifacts, which makes them an exciting prospect for clinical application; however, they often exhibit poor optical efficiency. They also require a trade-off between spatial and spectral resolution, although this is less problematic for hemoglobin measurements, in which the target spectra are well characterized, and extensive evidence exists for the application of optical measurements. Nonetheless, optimization of the target wavelengths for snapshot imagers can substantially improve their performance.^166^^,^^184^^–^^187^

In addition to these intensity-based imaging methods, spatial frequency domain imaging (SFDI) can be used to interrogate hemoglobin-related biomarkers by exploiting modulated illumination of the tissue. SFDI typically uses sinusoidal patterns and extracts the demodulation of these patterns reflected from tissue to calculate absorption and scattering properties; ^123^^,^^188^ low frequencies are more sensitive to absorption whereas high frequencies are more sensitive to scattering.^123^ Multiwavelength illumination^189^ can then be used to calculate hemoglobin content and oxygenation,^190^^,^^191^ e.g., in monitoring of peripheral circulation and vascular diseases,^192^ for which there are FDA-cleared devices, as well as ulcers,^193^ burns,^194^ or tumor margin detection.^195^^,^^196^ SFDI provides relatively high-resolution images, but conventional methods can be sensitive to motion artifacts^188^ and computational processing can limit the rate of image display. More recent reports have shown that it is possible to overcome these limitations using single snapshot of optical properties methods, which can achieve video-rate imaging.^197^

### 4.3 Analysis

Two- or three-wavelength imaging methods may be viewed qualitatively and interpreted by the operator, as in narrowband imaging, or processed to output quantitative THb or ${\mathrm{sO}}_{2}$ biomarkers in a manner similar to pulse oximetry, through calculation of image ratios and calibration of the results. For spectral imaging methods, analysis can be time-intensive due to the large amount of data collected, which can be problematic for clinical translation in which the real-time display of biomarker data is often desired. Analysis methods vary depending on the biomarkers targeted and the type of tissue imaged from the simplest techniques such as linear spectral unmixing^198^^,^^199^ to more complex methods such as multivariate analysis and machine learning.^26^^,^^72^^,^^164^^,^^165^ Linear spectral unmixing determines the type and concentration of chromophores present based on input reference spectra for oxy and deoxy hemoglobin, from which oxygen saturation can be calculated.^76^^,^^170^^,^^200^^,^^201^ Spectral signatures can also be used directly in classification of disease status, e.g., cancer.^171^^,^^181^^,^^202^^,^^203^ Sometimes, a combination of classification and unmixing techniques can produce the optimal results, allowing for data corrections to be applied in certain tissue types.^26^^,^^76^^,^^166^^,^^170^ Similar methods are also used in depth-resolved imaging, but data may need to be corrected based on the imaging depth and the associated level of optical absorption and scattering. Machine learning methods have shown promise in this regard, enabling more accurate determination of hemoglobin oxygenation, particularly at depth, than classic linear spectral unmixing.^204^

### 4.4 Limitations

Imaging tools for the assessment of hemoglobin can be subject to the same limitations as pulse oximetry. In addition, constraints that presently prevent clinical adoption of reflectance-based spectral imaging include cost, reliability of THb and ${\mathrm{sO}}_{2}$ measurements, clinical evidence for sensitivity and specificity of THb and ${\mathrm{sO}}_{2}$ in the diseases shown to be of interest in small-scale studies, and the need to process data in real time.^2^^,^^71^^,^^167^ These challenges are common in the clinical translation of optical imaging biomarkers,^2^ though fortunately, in the case of hemoglobin biomarkers, many devices have already navigated the pathway to the clinic, enabling initial studies to be undertaken.

## 5 Depth-Resolved Imaging

**Fig. 4:**
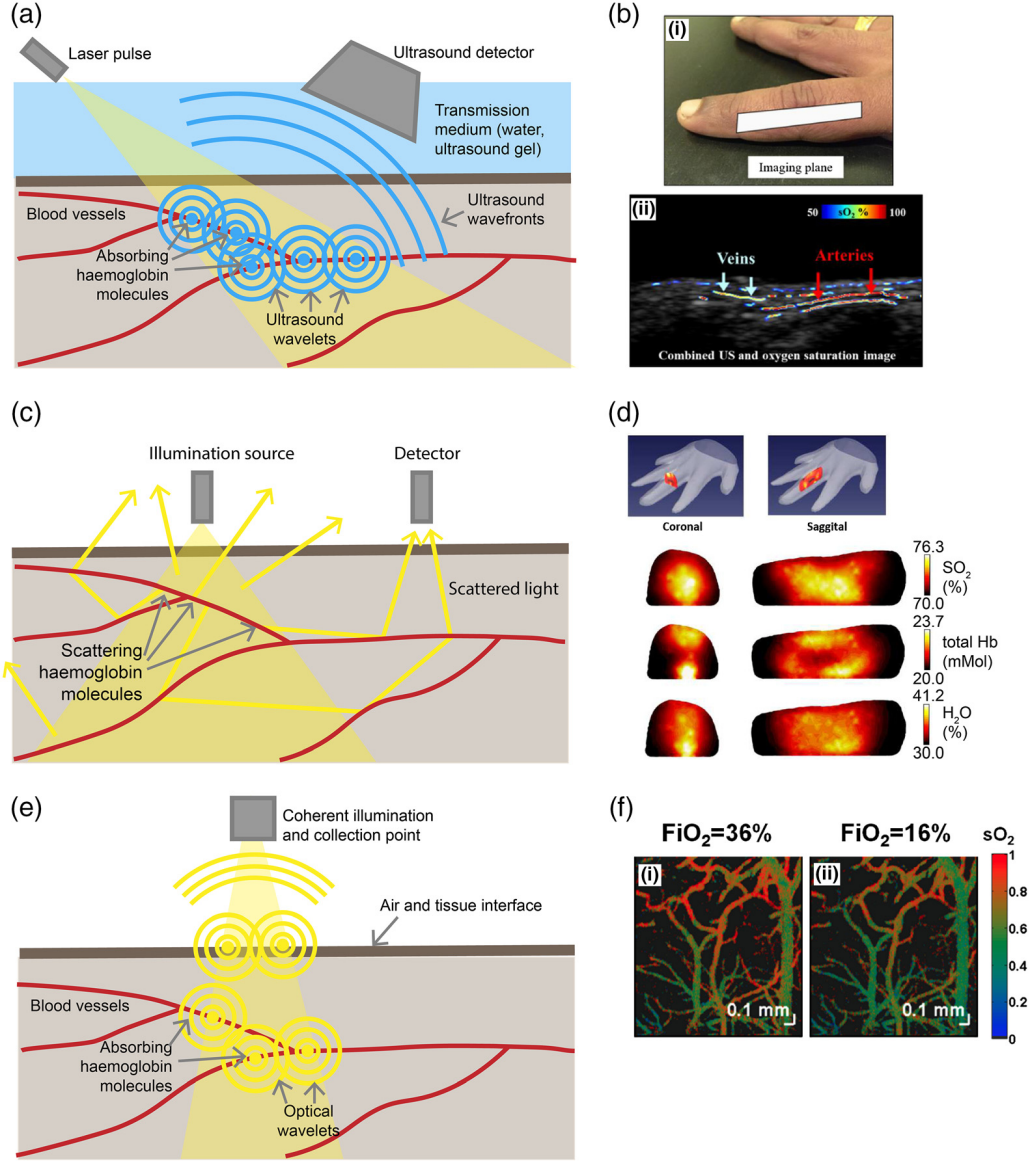
Principles of depth-resolved imaging. (a) In photoacoustic imaging, the absorption of light pulses generates a broadband acoustic wave detected at the tissue surface by an ultrasound transducer. (b) Photoacoustic imaging of oxygenation of the finger in combination with ultrasound to image the veins and arteries. Reproduced with permission from Ref. 205. (c) In DOI (and DCS techniques), illuminated light is scattered in tissue collected by an offset optical detector at the tissue surface. (d) DOI data acquired from the human finger is processed to quantify oxygenation, hemoglobin concentration, and water. Reproduced with permission from Ref. 206. (e) In OCT, coherent light illuminates the tissue, and the light that reflects at interfaces is collected and combined with a reference arm, so interference occurs; from this interference, depth-resolved images of the absorption and scattering properties of tissue can be resolved. (f) Oxygen resolved spectroscopic OCT on mice brains illustrating how the fraction of inspired oxygen (${\mathrm{FiO}}_{2}$) affects the oxygenation of the arteries and veins in the brain. Reproduced with permission from Ref. 207.

A key limitation of pulse oximetry and reflectance-based imaging is their inability to provide depth-resolved imaging of hemoglobin biomarkers. Two modalities are available to determine THb and ${\mathrm{sO}}_{2}$ in tissue up to and beyond depths of 1 cm: photoacoustic imaging (PAI) and diffuse optical imaging (DOI); both have been evaluated in clinical trials and are at different stages of clinical adoption. PAI exploits the generation of acoustic waves by the absorption of pulsed light by hemoglobin to create deep tissue volumetric maps using pulsed illumination and ultrasound detectors [Fig. 4(a)].^86^^–^^88^ DOI measures the properties of light scattering in tissue to generate absorption maps using synchronized illumination and photodiode-based detection [Fig. 4(b)].^124^^–^^126^ At more restricted depths, below 1 cm, spectroscopic optical coherence tomography (OCT) is also applicable, combining broadband illumination with spectrally-resolved interferometric detection. Although these methods have been widely explored in the clinical research setting, they are only just beginning to find routine application in the clinic for patient management.

### 5.1 Photoacoustic Imaging (PAI)

#### 5.1.1 Clinical research studies

**Fig. 5:**
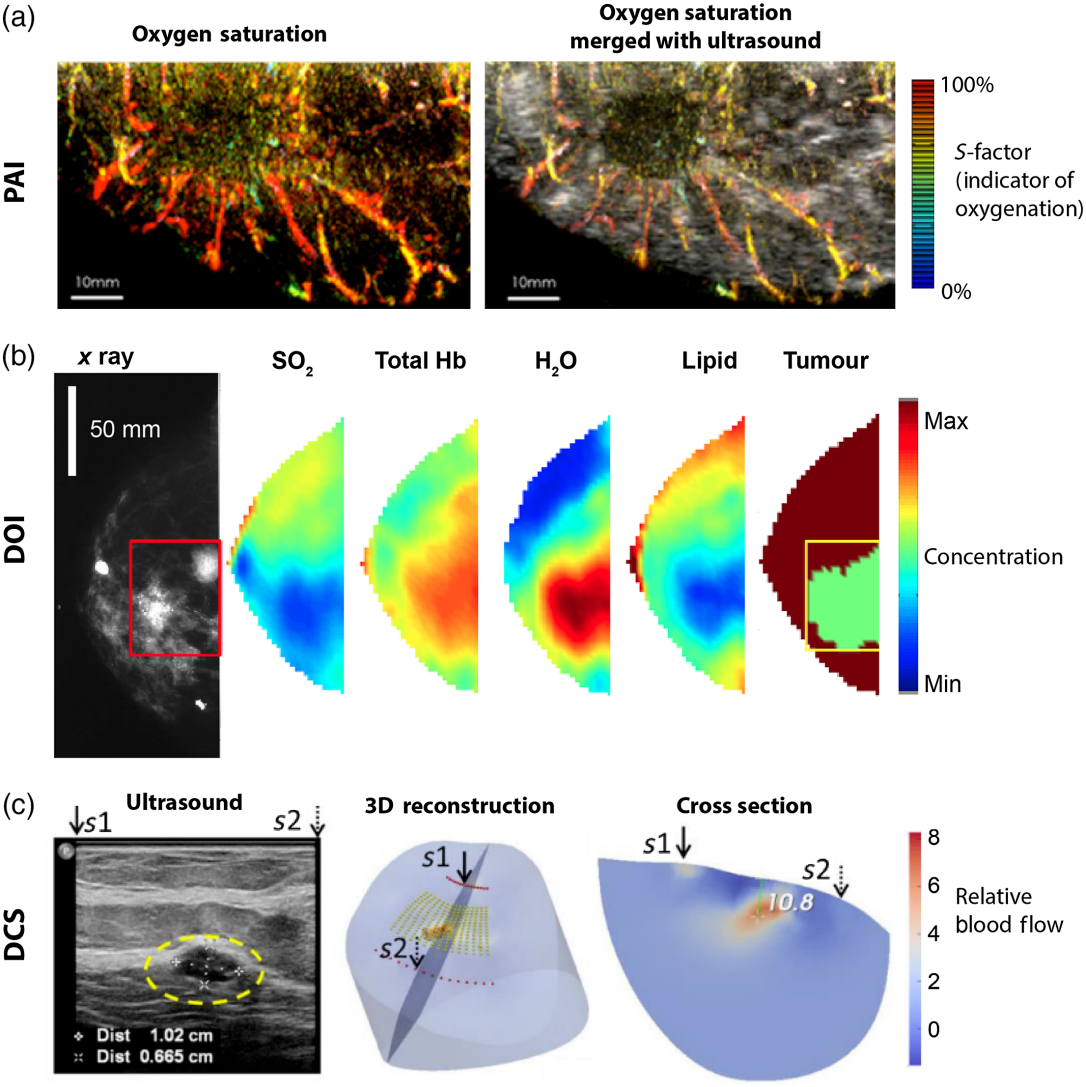
Tomographic imaging of the human breast for cancer detection (a) PAI of ${\mathrm{sO}}_{2}$ in breast with infiltrating ductal carcinoma (IDC); S-factor was defined to account for system accuracy and fluence compensation. Reproduced with permission from Ref. 210. (b) DOI of breast IDC (indicated by the red box) resolves ${\mathrm{sO}}_{2}$, THb, $H_{2}O$, lipid, concentrations of which serve to highlight the tumor. Reproduced with permission from Ref. 211. (c) DCS of blood flow relative to an ultrasound image of low-grade carcinoma; the tumor is circled in yellow. These images are referenced to positions s1 and s2 to compare the ultrasound, 3D reconstruction, and cross-section. Reproduced with permission from Ref. 212.

By far, the most explored clinical PAI application is human breast cancer detection, extensively reviewed elsewhere,^208^^,^^209^ due to the enhanced angiogenesis of breast cancers compared with background breast parenchyma and the scalability of PAI geometry allowing for a broad view of the area [Fig. 5(a)].^86^^,^^89^^,^^208^^,^^209^^,^^213^^,^^214^ Multicenter clinical trials have recently been concluded covering >2000 women; these established the ability of PAI to increase the specificity of ultrasound imaging using a real-time map of relative Hb and ${\mathrm{HbO}}_{2}$.^213^^,^^215^ PAI has also been explored in other cancer types, considering that neoangiogenesis is a hallmark of cancer, including thyroid,^216^ prostate,^215^ and melanoma among, others.^217^ Beyond applications in cancer, PAI has found a wide range of potential applications in which depth-resolved information is required, e.g., in endoscopic procedures;^90^^,^^91^ for evaluation of inflammation, such as arthritic joints,^88^^,^^92^ foot ulcers,^93^ and Crohn’s disease;^218^ vascular imaging;^94^ and for the guidance of interventional procedures, such as in fetal placentas.^95^

#### 5.1.2 Technology

Pulsed illumination is required to excite PAI signals. Typically, tunable pulsed lasers with nanosecond pulse durations have been used; however, pulsed laser diodes and LEDs have emerged recently as viable alternatives.^219^ The generated acoustic waves are detected by ultrasound transducers, which may be single-element, linear, or curvilinear arrays or in a spherical arrangement, depending on the system’s geometry. The type of transducer and associated center frequency or bandwidth is usually governed by the application, depending on the absorber size, laser pulse width, and required imaging depth.^220^ PAI can be deployed in different geometries, including tomography, mesoscopy, and microscopy. Tomography systems have found the most widespread clinical application as they provide an adequate field of view and spatial resolution for imaging of hemoglobin in deep tissue such as the breast; mesoscopy systems have also been applied clinically to visualize vascular network architectures in the skin given their limited penetration depths.^221^

#### 5.1.3 Analysis

The acoustic wave generated in response to pulsed optical illumination depends on the absorption properties of tissue according to

$$p_{0}(z)=\Gamma \mu_{a}F_{0}e^{-\mu_{0}z},$$ (13)

where $p_{0}$ is the initial pressure, Γ is the Gruneisen parameter, $F_{0}$ is the initial fluence, $\mu_{0}$ is a constant, and z is the depth of the tissue.^96^ PAIs are reconstructed using a range of beamforming methodologies, akin to ultrasound imaging.^94^^,^^96^ 3D tomographic images can be reconstructed by combining the temporal and spatial information collected, which is often achieved analytically using a simple back-projection inversion or numerically using model-based methods.^96^ Images reconstructed from data acquired at several wavelengths can then be subjected to the same multivariate analysis methods described in Sec. 4 for spectral unmixing. However, frame-to-frame coregistration may be needed to avoid spatial or spectral corruption due to motion.

#### 5.1.4 Limitations

The attainable depth of PAI depends on the optical and acoustic attenuation of the sample. In soft tissue, acoustic attenuation scales as a function of ultrasound frequency, so at low frequencies of a few MHz, optical attenuation tends to dominate and is the constraining factor for imaging depth.^96^ For spatial resolution, the constraining factor is the bandwidth of the acoustic wave, usually limited by the acoustic attenuation of soft tissue and the frequency response of the detecting transducer.^97^ The latter is particularly important for imaging more superficial features when the bandwidth of the signal can extend to 100 MHz and beyond, for which there is a limited availability of high-performance transducers. The maximum acoustic frequency transmitted decreases with depth, meaning that typically systems that operate at higher penetration depths have lower spatial resolutions than those designed for shallow imaging.^97^

A key challenge for PAI is biomarker quantification. During reconstruction, a number of assumptions are made; these include the speed of sound in tissue, transducer impulse response, detection bandwidth, and continuous sampling.^96^ If these assumptions break down, for example, due to heterogeneities in tissue due to air cavities, there will be distortions in the image. Furthermore, when evaluating biomarkers such as THb and ${\mathrm{sO}}_{2}$ the nature of light propagation in tissue can lead to distortions in the spectral properties of the illumination as a function of depth. Although some methods have been explored to compensate for such “spectral coloring,”^94^^,^^222^ they often break down in the complex scenarios found in human tissue and have yet to be validated in a clinical setting. Finally, as with all hemoglobin sensing and imaging methods, calibration of the extracted biomarkers is vital. PAI calibration and clinical quality assurance methods are still under development, particularly through a community-led effort.^98^

### 5.2 Diffuse Optical Spectroscopy and Imaging

#### 5.2.1 Clinical research studies

**Fig. 6:**
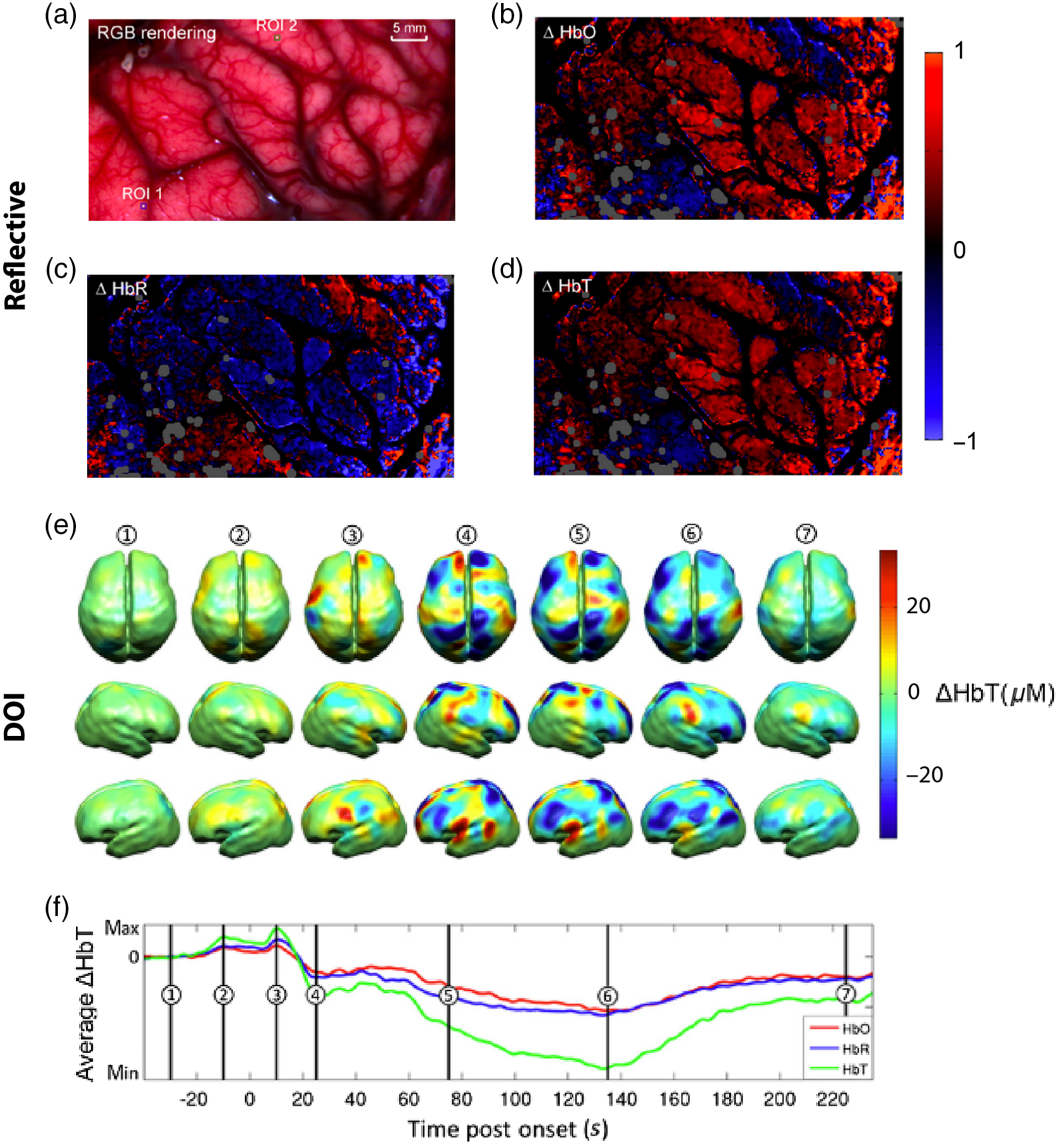
Hemoglobin imaging of the human brain. (a)–(d) Reflectance spectral images of the brain in an adult undergoing epileptogenic tissue resection (a) reference RGB rendering, (b) change in oxygenated hemoglobin over a single timeframe, (c) change in deoxygenated hemoglobin over a single timeframe, and (d) change in total hemoglobin over a single timeframe. Reproduced with permission from Ref. 76. (e), (f) DOI of a neonate during a seizure: (e) changes in HbT concentration mapped throughout the onset of a seizure and (f) average changes in Hb, ${\mathrm{HbO}}_{2}$, and tHb postonset of the seizure. Reproduced with permission from Ref. 223.

Diffuse optical spectroscopy (DOS) is commonly referred to as near-infrared spectroscopy (NIRS) because it uses light in the near-infrared range; the term functional NIRS (fNIRS) is also commonly used but is usually restricted to applications monitoring functional responses to stimuli in the brain via neurovascular coupling. Quantifying and monitoring changes in oxygenation of blood in the brain has found many applications that range from understanding seizures^223^ to detecting brain damage.^7^ Unlike reflectance hemoglobin imaging [Figs. 6(a)–6(d)], in which an open cranium is required (see Sec. 4), fNIRS typically achieves imaging depths of up to 15 mm through the skull [Figs. 6(e) and 6(f)], which covers the outer cerebral cortex in healthy adults.^127^^,^^223^^–^^225^ fNIRS imaging of the brain to identify intracranial hematomas due to brain trauma has been clinically approved by the FDA recently; however, it has yet to be widely deployed in clinical settings.^7^

Similar to PAI, DOI has also been deployed in clinical trials to detect cancer, particularly in the breast^226^^,^^227^ [Figs. 5(b) and 5(c)] and thyroid,^127^ where it has also been used to monitor response to therapy. DOI tends to be lower in spatial resolution than PAI (Fig. 5), but it can often resolve other biomarkers in addition to hemoglobin. PAI typically has a lower temporal resolution compared with DOI.^228^^,^^229^ In addition, DOI has been used to image muscle tissue such as the forearm, peripheral tissue, and joints.^127^^,^^225^ Several reviews have been published illustrating the importance of DOI.^230^^–^^233^

#### 5.2.2 Technology

DOI techniques typically use near-infrared light in the 650 to 900 nm range.^127^^,^^225^^,^^228^^,^^230^ Below 650 nm, light can experience poor penetration due to the absorption of hemoglobin in superficial tissues, whereas above 920 nm, water absorption comes to dominate.^225^ Most DOI and fNIRS systems use two (or more) different wavelengths, one that is in the lower range below the NIR isosbestic point, such as 680, 695, 705, or 730 nm, and another that has a longer wavelength, such as 830 or 850 nm. The number of light sources and detectors varies depending on the system from 1 to >48, with some designed to allow for more light sources and detectors to enable customization of spatial resolution according to the target application.^228^^,^^234^

DOS is similar to DOI but provides single-point measurements of tissue properties such as hemoglobin and blood oxygen saturation similar to pulse oximetry.^235^ There are three main modalities of DOS/I: continuous wave (CW), time-domain (TD), and frequency-domain (FD) systems.^127^^,^^225^^,^^228^ CW systems detect changes in the intensity of illumination, making them relatively simple and inexpensive.^236^ Slight changes in surface coupling can affect the intensity measurements, resulting in poor reproducibility unless well controlled. TD systems correlate the time between emission and detection of photons to measure the photon flight time. Tissue scattering determines photon flight time, whereas absorption determines the overall intensity of photons reaching the detector. FD systems are based on similar principles to TD systems but instead measure the phase shift of the incoming light. Frequency domain systems are significantly less expensive than TD systems due to lower costs for the sensors and detectors. TD and FD systems can distinguish contaminating signals due to background illumination because these signals will be uncorrelated. Because TD and FD systems can separate out the effects of scattering and absorption, these techniques can quantify absolute concentrations of hemoglobin and its oxygenation, whereas CW systems typically measure the relative change in concentration but not the absolute quantities.

DOI can be deployed in different geometries, either topographic, with imaging of a single plane with limited depth information, or tomographic (DOT), including depth resolution, which allows for full 3D reconstruction of the pertinent properties of tissue. In transillumination techniques, the illumination and detection are on opposite sides of the tissue being imaged; however, this is limited to body parts with small radii. Measurements at multiple and overlapping source–detector separations can be used to create depth measurements and reconstruct a 3D image. In tomographic systems, the illumination and detection sensors are placed on the available surface to simultaneously measure the changes in illumination throughout the sample.

Finally, it is worth noting that diffuse correlation spectroscopy (DCS) is similar to DOI and DOS, but it uses an autocorrelation function through a combination of hardware and software to measure an index of blood flow in tissue [Fig. 5(c)].^128^ Due to the similarities in apparatus, sometimes DCS is combined with DOI or DOS systems^128^ to provide a spatially-resolved indication of blood flow as a complementary biomarker to THb or ${\mathrm{sO}}_{2}$.^128^ Blood flow in tissue can also be detected using laser speckle contrast imaging (LCSI), which looks at fluctuations in the speckle pattern reflected from tissue to determine the flow rate of the blood.^237^^,^^238^ Laser Doppler flowmetry (LDF) finds the flow rate and concentration of blood by quantifying the Doppler shift that causes spectral broadening of reflected light. ^238^^,^^239^ All three of these techniques can resolve flow rate, but they typically have relatively low spatial resolutions or are confined to single-point measurements. Recent advances, particularly in LCSI, are now reaching near real-time operation at higher spatial resolutions, benefitting from increased computing power available in portable systems.^240^

#### 5.2.3 Analysis

The analysis for DOI depends on the type of imaging system used. The measured signals can be converted into optical absorption maps by understanding the transport of light in tissue, which can be modeled using the radiative transport equation (RTE)^127^^,^^225^ or Monte Carlo methods. The RTE is an analytical approach that approximates Maxwell’s equations in diffuse media, assuming a constant refractive index. Solving the RTE is computationally expensive,^225^ but it can be sped up by exploiting symmetries or by making approximations. For example, the diffusion approximation assumes isotropic scattering,^127^ so it can be applied only in diffusive tissues; it does not work well in nondiffusive tissue, such as the cerebrospinal fluid that surrounds the brain or in anisotropic media, such as the skin and nervous system, where prior information is required to reach a solution. Numerical techniques, such as the finite element method, finite difference method, finite volume method, and boundary element method, can be used to solve the diffusion approximation. The use of prior information such as MRI, CT, or other imaging techniques can vastly improve DOI resolution by reducing the number of assumptions about the tissue structure that are made.^225^

Light transport can also be understood by forward modeling the propagation of light using Monte Carlo methods to calculate the propagation of photons through media. The accumulated forward modeling statistics can then be analyzed with respect to real data from DOI systems to address the inverse scattering problem for various anisotropic scattering media.^225^ Monte Carlo methods have traditionally also been computationally expensive, but they are now reaching higher speeds with deployment on GPU and cloud-based servers.^241^

#### 5.2.4 Limitations

DOI exploits the scattering properties of tissue, unlike other imaging techniques that are hampered by light scattering. Despite this, DOI still has limited spatial resolution (on the order of 1 mm^230^) and often requires anatomical priors from another modality such as MRI or CT for analysis, which constrains applications^127^^,^^225^^,^^230^^,^^235^ and adds cost and complexity to high-resolution DOI systems.^225^ Standardization of DOI systems for clinical translation is ongoing, e.g., through a project to characterize DOI systems using phantoms,^242^ particularly for breast cancer detection.^243^

### 5.3 Spectroscopic OCT

#### 5.3.1 Clinical research studies

While OCT is primarily deployed for structural imaging, its spectrally-resolved detection can be harnessed for angiography and oximetry *in vivo*, albeit not yet in clinical practice. OCT is typically implemented in the NIR for structural assessment due to its greater penetration through tissue and availability of light sources with suitable coherence. Extraction of blood ${\mathrm{sO}}_{2}$ from OCT measurements in human retinal vasculature was first demonstrated in the NIR (800/850 nm),^244^ but it had limited precision due to weak absorption in this range. The development of visible OCT systems improved the available signal-to-noise ratio and hence precision, enabling measurements from single erythrocytes^110^^,^^111^ and high-resolution capillary oximetry in 3D.^112^^,^^113^^,^^245^ Advances in reconstruction algorithms and high-speed instrumentation have improved OCT angiography to a point at which it has found clinical use for high contrast imaging of retinal and dermal vasculature.^246^^,^^247^ Visible OCT measurement of retinal ${\mathrm{sO}}_{2}$ has been tested in humans^114^ alongside angiography,^115^ but it is still in development. Laser exposure limits and natural aversion restrict the usable power level for visible OCT in the eye compared with clinical NIR OCT, but continuing improvements in OCT technology have allowed for high-quality imaging and ${\mathrm{sO}}_{2}$ measurement.

#### 5.3.2 Technology

OCT is a noncontact imaging modality that can be considered an optical analog of ultrasound imaging.^116^ The distinguishing feature of OCT is the use of low-coherence interferometry to decouple the lateral and axial imaging resolution: lateral resolution is determined by the numerical aperture of the focusing optic, whereas axial resolution is determined by the temporal coherence of the laser used for imaging. Scanning OCT systems operate in the Fourier domain,^248^ where a broad spectrum of light interrogates the tissue and is then collected through spectrally-resolved detection for postprocessing and image reconstruction by an inverse Fourier transform. Within this class, there are two major varieties: spectral-domain OCT (SD-OCT), which uses broadband illumination and parallel detection with a spectrometer, and swept-source OCT (SS-OCT), which uses a high-speed spectral scanning source with single-channel detection for temporally-resolved spectral acquisition.

Recent advances in SS-OCT laser technology, including the tunable vertical-cavity source emitting laser, have enabled the realization of high-speed, robust, and compact instruments, which leads to greater imaging depths. Current SS-OCT systems operate exclusively in the NIR range due to swept laser availability; for imaging performance (and potential Hb oximetry) in the visible domain, SD-OCT systems using a visible spectrometer are required. In recent years, the high power and spatial coherence of newly available supercontinuum lasers have enabled good-quality visible OCT imaging in research systems,^117^^,^^249^^,^^250^ which could enable future developments toward clinical translation.

#### 5.3.3 Analysis

The raw data for SD-OCT are acquired from a high-speed spectrometer with line readout synchronized to a scanning mechanism, allowing for the mapping of each spectrum to a spatial position. The spectrum is normalized, filtered, and converted to a spatial reflectance profile, known as an A-line, through an inverse Fourier transform. For oximetry, the SD of this analysis must be narrowed, typically through a windowing function; although full spectral resolution of the OCT volume may be realized through the application of the short-time Fourier transform, the dependence of axial resolution on spectral bandwidth poses a necessary trade-off between spectral resolution and axial resolution in the spectroscopic OCT image. For ${\mathrm{sO}}_{2}$ measurement and spectral-contrast angiography, spectral windows are chosen to maximize the contrast of hemoglobin, typically in the range of 550 to 650 nm.^112^^,^^251^ The spectrally-resolved total extinction coefficient is measured through depth fitting of the spectroscopic OCT A-line signal to the Beer–Lambert law, from which the relative contributions of Hb and ${\mathrm{HbO}}_{2}$ can be unmixed to determine ${\mathrm{sO}}_{2}$.

#### 5.3.4 Limitations

OCT is highly versatile, being deployed for imaging the inner walls of blood vessels and luminal organs,^252^^–^^256^ having an ultrawide field of view for scanning skin,^257^^–^^259^ and with corrective lenses, compensating for ocular refraction in the ophthalmologic clinic. Nonetheless, there are several fundamental issues that are limiting its general adoption. First, the scanned acquisition adds instrumental complexity and can produce motion artifacts in patients. Although this is addressed in full-field OCT systems, these typically do not work with rough samples. Next, the spectral resolution of OCT imaging determines the maximum depth range that can be imaged, an aspect referred to as the sensitivity roll-off. Finally, because OCT is primarily sensitive to singly-scattered photons in tissue, the penetration depth of imaging is restricted to 1 to 2 mm in most human tissues. For this reason, large-scale clinical deployment of OCT has largely been limited to ophthalmology, dermatology, and cardiology, but creative advances in OCT probe and capsule technology will allow for continued *in situ* exploration of hemoglobin-related biomarkers from OCT in disease pathology throughout the body.^256^^,^^260^^–^^262^

## 6 Summary and Perspective

Moving beyond pulse oximetry to exploit the optical absorption of hemoglobin in imaging applications has shown significant promise in the clinic, with both superficial 2D and depth-resolved 3D implementations described in this review. Two wavelength THb and ${\mathrm{sO}}_{2}$ imaging are already widely used in endoscopic and ophthalmic applications, respectively, and have reached large-scale clinical trials in depth-resolved PAI. Conversely, reflectance-based spectral imaging remains largely exploratory.

### 6.1 Trade Offs

Choosing the optimal hemoglobin imaging technique for a particular application involves consideration of several factors that often require trade-offs, including signal-to-noise ratio, spatial and temporal resolutions, target depth, and route to integration with existing clinical practice. Optical imaging techniques are ultimately restricted by the maximum permissible exposure at the illumination site, which places a fundamental limit on the signal-to-noise ratio available in the clinical setting. Some techniques are further restricted in the type of illumination used such as OCT, which requires coherent light, and PAI, which requires short, pulsed light; this can add to the complexity of safety considerations in the clinic.

Considering the factors of resolution and depth, reflectance-based imaging can achieve high spatiotemporal resolution in applications in which depth resolution is not vital, for example, with retinal, endoscopic, or intraoperative imaging. One could argue that depth-resolution will become increasingly prevalent with the emergence of more advanced solutions from spectroscopic OCT, PAI, and DOI, particularly as costs decrease. Nonetheless, depth-resolution typically implies a sacrifice of spatial or temporal resolution, which must be determined early in the discovery and development phase of the associated device. Furthermore, different approaches to achieving depth resolution have different strengths and weaknesses. PAI may suffer from shielding effects due to the absorption of overlying tissue and tends to resolve only larger vessels, whereas the use of multiple scattering by DOI enables better detection of capillary oxygenation, despite the overall poorer spatial resolution. This trade-off may explain why PAI has developed more rapidly in clinical translation for breast cancer detection, whereas DOI is more developed for imaging the brain. Given the common contrast source across many of the techniques described, it may also be desirable to combine multiple approaches in a single device for validation purposes or to provide views of the same tissue at different resolution scales, overcoming some of the limitations of the existing technologies.^263^ Combining two or more optical imaging techniques can be beneficial because they reduce the imaging limitations that arise as a result of a single technique. A good example of this is the enhanced perfusion oximetry system that combines diffuse reflectance spectroscopy with LDF to quantify blood oxygenation and flow rate for imaging of microvasculature and burns.^264^^–^^266^

### 6.2 Clinical Implementation

There are many factors to consider regarding the route to integration of new techniques into clinical practice. For example, if imaging is required to be non-contact, for example, in delicate targets like the eye, this may restrict the implementation of methods such as PAI or DOI, in which contact with the tissue is typically required. The pathway to clinical adoption may be smoother in the case of an existing optical imaging solution already being deployed. An obvious example is the large-scale deployment of OCT for ophthalmic applications, which provides a direct route for adoption of spectroscopic OCT in the community. Another factor in clinical translation is the assessment of the precision, accuracy, and bias of new biomarker measurements.^2^ These factors can be affected by both device operation and data interpretation, requiring standardization of data acquisition and careful consideration of any signal processing or analysis methods applied before presenting the data to the interpreter, whether this is a human or a machine. Because reflectance-based imaging techniques are usually less computationally intensive compared with the depth-resolved methods, the development of systems and software for real-time imaging is more easily attainable; this is seen most effectively in narrowband imaging of blood vessels in endoscopy.

Further clinical considerations arise later in the translational pathway when the question of biomarker efficacy in decision-making finally arises. Instrument prototypes are often used first in pilot clinical trials at a single clinical center to gather initial data for validation and may be subject to multiple design iterations at this stage. Having successfully passed the first translational gap, which may include CE marking or FDA approval for the device and multicenter clinical trials, the technique is then subject to advanced qualification and ongoing technical validation to determine clinical utility in the healthcare setting, whereby the measurement can be used in clinical decision making. These larger-scale clinical trials help to determine sources of variation that will influence the classification and diagnosis of disease, providing clinical evidence of the ability to change patient management. They also provide extensive reference data sets that can be used to improve interpretation, particularly when machine learning-based methods are involved.

Reflectance-based spectral imaging techniques are still largely in the earlier stages of development with first in-person trials, whereas fNIRS of the brain and PAI of the breast are being used in multiple centers as part of larger-scale clinical trials and DOI has found some level of adoption into the clinic.^71^^,^^87^^,^^208^^,^^228^^,^^231^^,^^267^ Data arising from these trials is extremely valuable and making annotated datasets open source for the community in the future will not only help accelerate the development of new algorithms but could also enhance our understanding of the biology of hemoglobin oxygenation in disease.

### 6.3 Disease Monitoring

Hemoglobin imaging methods could find further applications in monitoring disease, to detect treatment efficacy or disease relapse. The noninvasive nature of these techniques can allow for continuous or periodic monitoring, for which there are several excellent examples that have been highlighted. Pulse oximetry can be applied to a patient for long periods so that clinicians can observe if there are any changes to overall arterial oxygenation. DOI and NIRS also can be used longitudinally to monitor changes in brain function to assess if there is improved brain activity.^267^ Furthermore, periodic monitoring of diseases such as scleroderma using nailfold capillaroscopy can indicate progression in the stage and severity of the disease, which may influence the treatment provided or indicate if further medical intervention is required.

Periodic monitoring can be applied in the short term, for monitoring wound or burn healing, or in the longer term in the context of endoscopic cancer surveillance in at-risk patient groups such as those with Barrett’s esophagus that are at increased risk of developing cancer. The frequency of monitoring applied is determined by the disease being observed and the rate at which change is expected, as well as by the training required for instrument use and data interpretation. For example, applying pulse oximetry is relatively quick with nonspecialists able to do it, and some more straightforward versions of capillaroscopy can be done using handheld devices. Conversely, endoscopies, OCT, DOI, and NIRS typically require training of specialist operators; hence it can be more expensive to conduct the procedures, meaning they are typically used for less frequent monitoring.

### 6.4 Outlook

The acceptability and relevance of new hemoglobin sensing and imaging technologies to clinicians will be driven by various factors, including cost, complexity, and physical size of the systems, as well as the ease of use and data interpretation. The commonplace use of pulse oximetry means that the clinical community is already well aware of the use of systemic ${\mathrm{sO}}_{2}$ as a disease biomarker. Ongoing technological developments that lead to miniaturization of light sources, optical components, and cameras, as well as decreasing their cost, mitigate some of the technical limitations highlighted in this review. Advances in image processing, including convolutional neural networks, promise to aid in distilling rich datasets to actionable clinical information, enabling imaging systems to be more easily integrated into clinical care. Research questions remain regarding the sensitivity of hemoglobin sensing techniques in diverse populations and the diagnostic power of hemoglobin-derived biomarkers in the wide array of disease presentations included in this review, which will only be answered through comprehensive clinical trials. Hemoglobin imaging techniques add a new dimension of knowledge in a range of clinical settings, from capillaroscopy and endoscopy to intraoperative imaging; emerging technologies are well placed to further enhance these areas of existing clinical practice, but are also likely to contribute to the decentralization of healthcare to tertiary care centers and through the deployment of wearable technologies for self-monitoring in the home.
